# Evaluating AI-generated curriculum designs in vocational education: Evidence from a No-AI diagnostic assessment

**DOI:** 10.1371/journal.pone.0352555

**Published:** 2026-08-14

**Authors:** Zheng Liu

**Affiliations:** Henan Forestry Vocational College, Luoyang, Henan, China; Golestan University, IRAN, ISLAMIC REPUBLIC OF

## Abstract

This study examines how vocational students evaluate AI-generated curriculum designs after using generative artificial intelligence to support course-planning tasks. It reports an interpretive single-case study of a Study Tour Curriculum Design course in a Chinese vocational college. Data comprised course task briefs, the instructor’s reflective teaching memo, and a closed-book handwritten diagnostic assessment completed by 39 second-year students without AI access. The diagnostic assessment yielded 182 substantive diagnostic claims, which were analysed through thematic and directed content analysis, with students’ complete responses also assigned holistic written judgement levels. Students could state broad criteria for judging AI-generated work, including accuracy, feasibility, learner appropriateness, safety, and the need for human revision. Their applied diagnostic performance was uneven. Most identified visible implementation problems such as learner mismatch, time pressure, site overload, and safety insufficiency, while fewer diagnosed deeper issues of curriculum logic, assessment operationalisability, fact verification, and author responsibility. The study identifies this pattern as a stated-applied judgement gap. The findings suggest that no-AI diagnostic assessment can make students’ judgement of AI-generated vocational work visible and can help teachers identify where AI-assisted task completion has not yet developed into stable professional judgement. The study offers a transparent classroom case of how AI-generated outputs can be evaluated in vocational education, while recognising the limits of a single-case design.

## 1. Introduction

Generative AI has changed the evidentiary status of task completion in vocational education. In text-based and design-oriented courses, students can obtain complete-looking reports, plans, scripts, proposals and curriculum designs within seconds. Under these conditions, a finished assignment becomes a weaker indicator of learning because it may show access to AI-assisted production while revealing less about students’ capacity to judge professional adequacy, identify missing decisions and take responsibility for feasibility, safety, accuracy and educational value.

This evidentiary problem became visible in a Study Tour Curriculum Design course at a vocational college in central China. During task explanation, several student groups used generative AI tools to produce curriculum design drafts before the teacher had finished explaining the requirements. The drafts contained expected sections, fluent language and apparently coherent activity sequences, yet the professional reasoning behind them remained weak. Students had produced the outward form of vocational work without fully engaging in the judgement required to make that work professionally usable.

This problem matters in vocational programmes because many tasks are designed to approximate workplace practice. Students are expected to develop occupationally relevant judgement by interpreting constraints, producing work and revising it against professional standards. When AI can generate the surface structure of such work almost instantly, the pedagogical problem shifts from producing an acceptable draft to diagnosing and improving an AI-generated draft through the standards of a field.

This study examines that problem through a Study Tour Curriculum Design course taught to 39 second-year vocational students in China. Study tour curriculum design is used as a domain-specific vocational task because it requires students to connect learner characteristics, site resources, learning objectives, activity design, safety management, assessment and evidence of learning. It is also highly susceptible to AI-assisted production because the expected document form is structured, text-heavy and easily imitated. The course therefore provides a useful setting for examining how students learn to judge AI-generated professional work when AI can already generate its textual form.

The teacher’s response centred on diagnosis and revision. Students began with AI-assisted drafts, while the teacher circulated among groups, identified missing professional decisions and directed students toward more workable revisions. This paper conceptualises that role as teacher calibration: a pedagogical role in which a domain-expert teacher makes professional judgement criteria visible by diagnosing what is missing, weak or misaligned in students’ AI-assisted work. In this case, calibration concerned learner-stage appropriateness, objective-task alignment, site feasibility, learning evidence, assessment operationalisability, safety and factual verification.

The study addresses three research questions:

RQ1. How was teaching reconfigured when generative AI made complete-looking vocational task outputs easy to produce?

RQ2. What patterns of diagnostic judgement did vocational students display when evaluating an AI-generated curriculum design without AI access?

RQ3. What does the relationship between students’ stated AI judgement criteria and their written diagnostic performance reveal about AI-assisted vocational learning?

The study focuses on three empirical and analytical issues. First, it examines whether students who can articulate responsible AI-use criteria can also apply those criteria when diagnosing an AI-generated task output. Second, it examines how a no-AI diagnostic assessment can separate fluent AI-assisted task completion from students’ independent evaluation of professional adequacy. Third, it analyses teacher calibration as a classroom process through which a domain-expert teacher made professional judgement criteria visible during the revision of AI-assisted work.

The claims are deliberately bounded. This is an interpretive single-case study of one course, one teacher and one cohort of vocational students. The diagnostic task was a structured elicitation device, not a psychometric measure. The study does not claim causal improvement in AI critical competency. Its purpose is to report how students’ judgement of AI-generated vocational work was elicited and analysed in a real classroom case.

## 2. Literature review

### 2.1 Generative AI and the evidentiary problem of vocational task completion

Task-based learning is important in vocational education because professional capability is developed through purposeful activities that approximate occupational practice. In competency-oriented curricula, students learn by interpreting task requirements, mobilising knowledge, making decisions, producing work and revising it against professional standards [1–3]. This logic assumes that completed work has a meaningful relationship to students’ reasoning and capability.

Generative AI unsettles this assumption. Large language model-based tools can produce complete-looking reports, plans and curriculum documents within seconds. Recent scholarship has raised concerns about AI-facilitated bypassing, cognitive offloading, surface compliance and the changing evidentiary status of student work [4–8]. When students can obtain a fluent draft before engaging deeply with a task, the final product becomes a less reliable indicator of what they understand or can do. This issue is especially consequential in vocational education, where written assignments often simulate professional deliverables and are used to infer occupational capability.

Student-facing studies also show that the educational value of generative AI depends on how learners engage with the tool. Students report benefits such as efficiency, idea generation and language support, while also raising concerns about accuracy, ethics and over-reliance [9]. A recent systematic review of generative AI in higher education similarly shows that students use these tools for content generation, learning support and evaluation, with learning outcomes shaped by task design and self-regulated use [10]. Research on generative AI and critical thinking further suggests that AI confidence may reduce users’ self-reported critical thinking effort during knowledge work [11]. Studies of AI and higher-order thinking in language learning indicate that AI use may create both opportunities and constraints for critical evaluation and independent judgement [12]. Research on AI adoption in self-regulated English learning also links learners’ engagement with AI to motivation and digital literacy [13]. Together, these studies support the need to examine students’ diagnostic judgement of AI-generated work as a distinct source of learning evidence.

Assessment responses to generative AI have included ethical incorporation, redesign frameworks and outcome-focused reviews [14,15]. Future-oriented work also calls for higher education research to examine how generative AI reshapes teaching, learning and assessment practices [16]. These responses identify broad directions, yet they do not fully resolve the problem of learning evidence in AI-pervasive vocational classrooms. This study argues that one observable site of learning is students’ diagnostic judgement of AI-generated work: their ability to identify what is professionally weak, missing or misaligned in an AI-produced output and explain how it should be improved.

### 2.2 AI-output judgement as professional capability

AI literacy has been conceptualised as a multidimensional construct involving knowledge of AI, use and application, evaluation and creation, and ethical awareness [17,18]. More recent frameworks distinguish broad AI literacy from situated AI competency, emphasising critical evaluation and contextual application [19,20]. Self-report and scale-development studies have also operationalised AI literacy across affective, behavioural, cognitive, ethical and competence dimensions [21,22]. However, much empirical work on AI literacy still relies on self-report or general competence measures, with less attention to how students judge AI outputs within specific professional tasks.

The growing literature on generative AI literacy reinforces the need for task-specific analysis. Recent scale-development research on GenAI literacy shows that users’ knowledge, evaluative awareness and responsible use can be treated as measurable educational constructs [23]. Studies of GenAI self-regulation further connect AI-supported learning with critical thinking and problem solving [24], while design work grounded in self-regulated learning shows that learner agency and instructional design shape how students engage with generative AI tools [25]. For vocational education, these capacities need to be examined through domain-specific work. In the present study, AI-output judgement refers to students’ capacity to evaluate whether an AI-generated professional document is feasible, safe, learner-appropriate, assessable and factually responsible.

Evaluative judgement offers a bridge between AI literacy and vocational capability. It refers to the capacity to make informed decisions about work quality [26,27]. In generative AI contexts, students must judge both their own learning processes and outputs produced through human-AI collaboration [28,29]. For vocational education, this judgement has a professional character: students must evaluate whether an output is safe, feasible, contextually appropriate, ethically responsible and aligned with field standards.

This study uses the term AI-output judgement to describe students’ capacity to evaluate AI-generated professional work through domain-specific criteria. In vocational curriculum design, these criteria include learner appropriateness, objective-task alignment, resource feasibility, task educational validity, evidence of learning, assessment operationalisability, safety and fact verification. This form of judgement is a professional capability because it concerns the quality and responsibility of work that may affect real learners, clients, users or workplace stakeholders. It also connects to professional judgement in vocational knowing: practitioners must interpret situations, weigh constraints and act responsibly under uncertainty [30].

### 2.3 Teacher calibration and diagnostic assessment

Generative AI also changes the role of teachers in vocational classrooms. Existing literature describes teachers in AI-integrated environments as AI curators, AI instructors, learning designers or human counterparts who provide judgement and contextual support [31,32]. Recent GenAI studies further show that AI-mediated classrooms continue to depend on teachers’ pedagogical decision-making and communicative guidance [33,34]. Teacher presence and self-determination-theory-based teacher support have also been associated with engagement, higher-order thinking and learning outcomes in GenAI-supported learning environments [35,36]. Scaffolding and feedback research further shows that teachers support learning by adjusting guidance to learners’ needs and directing attention toward improvement [37–39].

This paper develops the concept of teacher calibration to describe a more specific role in AI-pervasive vocational learning. Teacher calibration refers to the process by which a domain-expert teacher diagnoses the professional judgement missing from students’ AI-assisted work and makes the relevant criteria visible for revision. The teacher moves beyond improving text or providing general advice, identifying which professional decision has not yet been made, which contextual constraint has been ignored or which responsibility has been displaced onto a fluent AI draft.

Diagnostic assessment is one way to make this judgement visible. In AI-pervasive courses, a completed assignment may not show whether students can independently evaluate AI-generated work. A diagnostic task can ask students to identify problems, explain why those problems matter and propose revisions without AI access. It can serve as a structured elicitation device that reveals which professional criteria students can apply and where their judgement remains uneven.

The present study examines these issues in a Study Tour Curriculum Design course. Study tour curriculum design is a domain-specific vocational task because it requires students to integrate tourism resources, learner characteristics, curriculum objectives, field activities, safety arrangements and assessment evidence. It is also vulnerable to generative AI because the written format is structured and easily imitated. The course therefore provides a concrete setting for examining how teacher calibration and no-AI diagnostic assessment can support AI-output judgement in vocational education.

### 2.4 Theoretical positioning and research gap

This study integrates three strands of literature. AI literacy research explains why students need to understand, use, evaluate and act responsibly with AI systems [17–25]. Evaluative judgement and professional judgement research explain why the capacity to judge work quality is central to learning and professional practice [26–30]. Teacher support and feedback research explain how domain-expert teachers can make quality criteria visible and guide revision in complex learning tasks [33–39].

The resulting theoretical position treats AI-output judgement as a situated professional capability. In the present case, students were expected to judge an AI-generated study tour curriculum design through learner-stage appropriateness, objective-task alignment, site feasibility, learning evidence, assessment, safety and factual responsibility. Teacher calibration is positioned as the instructional process through which a domain-expert teacher makes these criteria visible during the revision of AI-assisted work.

The research gap concerns the evidentiary status of students’ judgement in AI-assisted vocational tasks. Existing studies have examined students’ perceptions of generative AI, AI literacy, self-regulated AI use and teacher support in AI-enhanced learning environments [9,10,23–25,33–36]. Less is known about how vocational students independently diagnose a flawed AI-generated professional output when AI access is removed. This study addresses this gap through a no-AI diagnostic assessment and a transparent coding framework.

## 3. Materials and methods

### 3.1 Research design and case context

This study adopted an interpretive single-case study design [40,41]. The case was bounded by one vocational course, one instructor, one cohort and one semester. This design was appropriate because the study aimed to develop a contextually grounded understanding of how generative AI reshaped task-based vocational teaching and how students’ diagnostic judgement of AI-generated work could be made visible.

The case was a Study Tour Curriculum Design course in a vocational college in central China. The course was part of a tourism management programme and required students to design educational field-learning activities for defined learner groups. The cohort consisted of 39 second-year vocational students. The instructor had 12 years of experience in vocational tourism education, curriculum design and related professional practice. Students frequently used Chinese AI tools such as Doubao, DeepSeek, Kimi and Yuanbao to generate draft curriculum documents. The instructor therefore reorganised classroom work around iterative diagnosis and revision. Students produced AI-assisted drafts, received feedback on missing professional decisions and revised their designs in relation to learner population, site feasibility, task design, learning evidence, assessment and safety.

### 3.2 Data sources

The study used three sources of material. The first was the documented sequence of course task briefs, which showed how task requirements progressively foregrounded professional judgement criteria. The second was the instructor’s reflective teaching memo, which recorded the pedagogical rationale for redesigning the course, observations of students’ AI-assisted drafting practices and reflections on teacher calibration. It was used as contextual material, not as independent evidence of student learning outcomes.

The third and central source was a closed-book handwritten diagnostic assessment administered at the end of the course. Students submitted their mobile phones before the session and wrote by hand for approximately 60 minutes without AI access. All 39 students attended and submitted substantively complete responses. The handwritten responses were transcribed into typed Chinese text and manually checked against the original scripts. Parts One and Two asked students to describe their AI use, distinguish between using AI and judging AI-generated content, and reflect on future human-AI task division. Part Three presented an AI-generated study tour curriculum plan and required students to identify five problems, explain why each was problematic and propose revisions. The full instrument is provided as Supplementary material appendix 1.

### 3.3 Data analysis

The analysis proceeded in three stages. First, responses to Parts One and Two were analysed thematically [42]. Coding focused on how students described AI use, what they said they checked in AI-generated work and how they imagined future human-AI professional task division.

Second, responses to Part Three were analysed through directed content analysis [43,44]. The unit of analysis was a distinct diagnostic claim, usually consisting of a problem, reason and proposed modification. The prompt invited 195 potential entries across 39 students. Repeated entries within the same response were merged, and entries without a substantive diagnostic claim were excluded, retaining 182 diagnostic claims.

The coding framework contained 12 categories developed from the course criteria, diagnostic prompt and literature on evaluative and professional judgement. These categories covered learner population appropriateness, site and resource selection, learning objective quality, task educational validity, operational feasibility, safety management, learning evidence, assessment operationalisability, fact verification, AI judgement metacognition, teacher scaffolding role recognition and curriculum author responsibility. Multiple coding was allowed.

Third, each student’s complete Part Three response was assigned a holistic written judgement level from 0 to 4. Level 0 indicated no substantive diagnostic engagement; Level 1 minimal or vague engagement; Level 2 recognition of visible implementation problems; Level 3 multiple substantive problems with some curriculum reasoning; and Level 4 integrated curriculum logic judgement across learner population, objectives, tasks, evidence, assessment, feasibility, safety and responsibility. These levels were interpretive categories, not psychometric scores. The coding framework and judgement-level criteria are provided as Supplementary material appendix 2.

Category frequencies and judgement-level counts were used only as descriptive summaries of the case. No inferential statistical tests, population estimates or psychometric claims were attempted.

### 3.4 Ethics and trustworthiness

This study analysed routine course-related written materials generated in a normal instructional context. The diagnostic assessment was a normal component of the course’s reflective learning activity, and no research-specific intervention beyond regular teaching was introduced. All participating students were second-year adult students. In the instructor’s normal teaching role, the author initially had access to student names on the handwritten scripts. Direct identifiers were subsequently removed and replaced with codes from S01 to S39 before analysis and reporting.

Formal ethics committee review was not obtained. The retrospective analysis used anonymised routine educational records, introduced no research-specific intervention, reported no grades or rankings, and involved no sensitive personal information or commercial interests. The author considered the analysis eligible for exemption under Article 32(2) of the 2023 Measures for Ethical Review of Life Science and Medical Research Involving Humans, which allows exemption for qualifying research using anonymised information data. This statement describes the basis used by the author and does not claim that an institutional committee issued an approval or waiver.

The case boundary is explicit; the assessment was conducted without AI access or mobile phones; original handwritten responses were preserved; transcriptions were manually checked; and English excerpts were checked against the Chinese originals. These procedures supported trustworthiness in qualitative case analysis [45]. Claims about student judgement are grounded in written assessment responses, while the instructor memo contextualises the pedagogical design. The study does not report individual grades, rank students or make claims about individual learning outcomes. This qualitative classroom case is reported in alignment with the Standards for Reporting Qualitative Research. The coding framework, judgement-level criteria and diagnostic instrument are provided as supporting information to support transparency and review.

### 3.5 AI-use disclosure

During manuscript preparation, the author used Doubao for limited English language polishing and expression refinement. The tool was used to improve grammar, sentence fluency and formatting consistency. It was not used to generate the research idea, design the study, collect data, code data, analyse data, interpret findings, draw conclusions or generate the supporting dataset. The author checked and edited all AI-assisted language suggestions against the original Chinese materials, coding records, cited literature and manuscript argument, and takes full responsibility for the accuracy and integrity of the submitted work.

## 4. Results

### 4.1 AI-generated surface completeness and the evidentiary problem of completed tasks

The course was reorganised after the instructor observed that several student groups could generate complete-looking curriculum design drafts before the task requirements had been fully explained. The drafts had formal completeness, with themes, objectives, activity procedures, assessment and safety notes. However, they often lacked the professional decisions needed to make a curriculum design workable in practice. This made task completion an unreliable indicator of vocational learning.

The instructor responded by shifting classroom work from first-draft production to diagnosis and revision. Students could use AI to generate initial drafts, but the main instructional work occurred after those drafts appeared. The instructor examined each AI-assisted draft and identified the most consequential missing judgement. Feedback focused on learner population, objective-task alignment, site feasibility, task validity, learning evidence, assessment, safety and factual accuracy.

This process is described here as teacher calibration. It involved more than correcting language or improving document structure. The teacher redirected students’ attention to professional criteria that were absent or weakly handled in AI-generated work. For example, when a group proposed abstract historical objectives for young learners, feedback focused on whether those learners could observe, compare or express anything at the site that would make the objective accessible. When activities looked coherent on paper, the teacher asked whether learners had a clear action, whether the activity could be completed in the available time and whether it would produce evidence of learning.

The calibration process was distributed and iterative. The teacher provided brief, situated interventions to different groups, then allowed students to revise and often reprompt AI with more specific guidance. Later simulation work reinforced the same pattern: plans that seemed coherent in writing sometimes failed during enactment because tasks lacked observation protocols, discussion questions were too broad or learner roles collapsed into passive listening. These problems returned students to the same professional criteria of learner action, objectives, learning evidence, feasibility and safety.

### 4.2 Stratified evaluative judgement in the no-AI diagnostic task

The no-AI handwritten assessment provided evidence of how students diagnosed an AI-generated curriculum plan independently. Each student’s complete Part Three response was assigned a holistic written judgement level from 0 to 4, considering the range of problems identified, explanation provided and connection to curriculum design logic.

Table 1 shows a stratified pattern. All 39 students made substantively meaningful diagnostic comments, and no student was placed at Level 0 or Level 1. Ten students were classified as Level 2 surface checkers. They identified visible implementation problems such as learner age mismatch, excessive site coverage, time pressure or vague safety requirements, while reasoning remained mainly local and practical. Seventeen students were Level 3 partial curriculum judges, identifying several substantive problems with some educational design reasoning. Twelve students were Level 4 curriculum logic judges, connecting visible problems to deeper misalignments among learner characteristics, objectives, learner action, learning evidence, assessment and responsibility.

**Table 1:** Distribution of written judgement levels in the No-AI diagnostic task (N = 39).

Written judgement level	Description	n	Proportion
Level 4: Curriculum logic judges	Identified systemic misalignments among objectives, tasks, learning evidence, assessment, learner population, and professional responsibility; demonstrated integrated curriculum design judgement	12	30.8%
Level 3: Partial curriculum judges	Identified multiple substantive problems and provided some educational design reasoning; judgement was substantive but uneven across categories	17	43.6%
Level 2: Surface checkers	Identified visible implementation problems such as time pressure, learner age mismatch, site overload, or safety insufficiency; reasoning remained mainly local and practical	10	25.6%
Level 1/0: Minimal diagnostic engagement	Provided vague, non-analytical, or substantively incomplete responses	0	0%

Across the 39 responses, 182 distinct diagnostic claims were retained for coding. A single claim could receive more than one code when it addressed multiple dimensions.

Table 2 indicates that students most frequently identified learner population appropriateness, task educational validity, learning objective quality, site and resource selection, operational feasibility and safety management. These categories were visible in the AI-generated plan because it required primary school third-graders to visit several historical-cultural sites, conduct interviews, discuss complex themes and produce outputs within 45 minutes.

**Table 2:** Problem categories identified in the No-AI diagnostic task.

Code	Problem category	Students identifyingcategory n (%)	Diagnostic claims
CJ1	Learner populationappropriateness	37 (94.9%)	83
CJ2	Site and resource selectionjudgement	33 (84.6%)	56
CJ3	Learning objective quality	34 (87.2%)	76
CJ4	Task educational validity	37 (94.9%)	102
CJ5	Operational feasibility	33 (84.6%)	54
CJ6	Safety managementadequacy	32 (82.1%)	60
CJ7	Learning evidence quality	24 (61.5%)	50
CJ8	Assessmentoperationalisability	12 (30.8%)	14
CJ9	Fact verification	13 (33.3%)	13
CJ10	AI judgement metacognition	11 (28.2%)	13
CJ11	Teacher scaffolding rolerecognition	22 (56.4%)	31
CJ12	Curriculum authorresponsibility	15 (38.5%)	19

*Note. One diagnostic claim could be assigned multiple codes; therefore, diagnostic-claim counts are not additive across categories.*

Less frequently identified categories included assessment operationalisability, fact verification, explicit AI judgement metacognition and curriculum author responsibility. Many students could identify that the plan was too difficult, crowded or unsafe. Fewer examined whether assessment criteria were observable, whether factual claims required verification or whether the human curriculum designer remained responsible for the professional consequences of using an AI-generated plan.

Student explanations show the difference between surface checking and integrated judgement. Safety provides one example. Some students wrote that safety requirements were too simple. A stronger response treated safety as a precondition for field implementation: “Safety requirements should be communicated before activities begin, not placed at the very end of the entire document… Content should be expanded to include warnings such as avoiding narrow paths and street stalls” [S05, Part Three]. A similar contrast appeared in responses to the photography task. Some students noted that third-grade pupils might be too young to take photographs independently. A stronger response connected the task to learning evidence: “Students only recording content does not mean they are internalising it… the teacher [should set] questions to prompt active response” [S11, Part Three].

### 4.3 The stated-applied judgement gap in students’ evaluation of AI-generated work

Parts One and Two showed that many students could articulate a distinction between using AI and judging AI. They described AI as useful for generating structures, drafting plans, organising information, refining language and producing formats, while stating that AI-generated work required human checking for factual accuracy, feasibility, learner appropriateness, safety and alignment with course requirements.

Several students expressed this distinction clearly. S36 wrote, “In Year One I could not distinguish AI content from real content and believed it without question. Now I can judge from the details” [Part Two]. S05 wrote, “I base my judgement on the historical knowledge I already have. For knowledge points I am unsure about, I search further to verify them” [Part Two]. The same student added, “Judging AI means making the generated content match real circumstances and making the plan genuinely implementable” [Part Two].

The diagnostic task showed that this stated awareness did not translate evenly into integrated performance. Students most consistently applied judgement to visible problems: learner age, task difficulty, time pressure, site overload and safety insufficiency. Deeper curriculum issues appeared less consistently. Fewer responses sustained attention to alignment among objectives, learner action, learning evidence and assessment. Some students could state that a goal was too broad or a task too difficult, while fewer explained how the task should be redesigned to produce observable learning evidence or how assessment criteria should correspond to intended outcomes.

This pattern reveals a stated-applied judgement gap in AI-assisted vocational learning. Students had developed a language of responsible AI use, but their written performance in curriculum logic diagnosis remained uneven. Their projections of future professional work showed the same boundary: AI could support structural generation, information aggregation, route planning, formatting and drafting, while humans should retain site-specific judgement, learner-appropriate task design, safety decisions, factual verification and educational responsibility. AI-output judgement was therefore visible, but its depth depended on whether students could apply professional criteria beyond the most obvious defects of the AI output.

## 5. Discussion

### 5.1 Students’ uneven diagnostic judgement of AI-generated curriculum designs

A central implication of this study is that generative AI creates an assessment problem concerning the difference between students’ stated judgement criteria and their applied evaluative performance. Many students could explain that AI-generated work should be checked for accuracy, feasibility, learner appropriateness, safety and contextual fit. Their no-AI diagnostic responses showed a more uneven pattern: visible implementation problems were identified more consistently than deeper curriculum issues involving objectives, learner action, evidence, assessment and author responsibility.

This stated-applied judgement gap matters because generative AI changes what completed assessment tasks can evidence. When students can produce complete-looking curriculum designs within seconds, a finished document may demonstrate access to AI-assisted production while offering limited evidence of students’ capacity to evaluate professional adequacy. The diagnostic task in this study made judgement visible by asking students to identify problems, justify why they mattered and propose revisions without AI access.

The finding extends current debates on evaluative judgement in generative AI contexts. It suggests that students’ ability to state responsible AI-use principles should be distinguished from their ability to enact those principles in concrete task evaluation. Assessment design therefore needs to include opportunities for students to demonstrate how they judge AI-generated outputs, especially when those outputs concern professional work with consequences for learners, sites, safety and evidence of learning.

Teacher calibration becomes important within this assessment problem. In the course examined here, the teacher’s feedback helped students move from accepting the surface completeness of AI-assisted drafts to recognising missing professional decisions. Calibration made evaluation criteria visible during revision and prepared students for the later diagnostic task, where the depth and limits of their AI-output judgement could be examined.

### 5.2 AI-output judgement as professional capability

The stratified diagnostic pattern suggests that AI-output judgement should be understood as a vocational capability. Students needed more than tool-use competence. They needed to judge whether an AI-generated professional output was feasible, safe, age-appropriate, educationally meaningful, assessable and factually responsible. This type of judgement concerns whether work can be responsibly used in practice.

The findings show that students’ AI-output judgement was uneven. All students identified some problems, and many noticed visible defects such as time pressure, learner mismatch, site overload and vague safety requirements. However, fewer connected these visible problems to deeper curriculum logic. Integrated judgement required students to examine relationships among objectives, learner action, learning evidence, assessment and responsibility. This distinction matters because professional work often fails at the level of alignment, feasibility and consequence, even when the document appears complete.

This finding extends AI literacy research by giving the evaluative dimension a vocational interpretation. In vocational learning, evaluation must be grounded in field standards. A student evaluating an AI-generated curriculum plan must ask whether real learners could complete the task, whether the site supports the intended learning, whether assessment criteria are observable and whether safety arrangements are adequate. AI-output judgement should therefore be taught and assessed through domain-specific professional tasks.

The study also contributes to evaluative judgement research. In AI-pervasive vocational education, the “work” to be judged increasingly includes AI-generated or AI-assisted outputs. Diagnostic tasks can make such judgement visible by asking students to identify problems, justify reasoning and propose revisions. Their value lies in revealing what criteria students can apply and where professional reasoning remains underdeveloped.

### 5.3 From declarative AI awareness to stable professional judgement

Whereas Section 5.1 treated the stated-applied judgement gap as an assessment-design problem, this section considers how that gap may emerge in AI-assisted vocational learning. The data suggest that students acquired a declarative language of responsible AI use before developing stable professional judgement. Many students could explain that AI-generated work should be checked for accuracy, feasibility, safety, learner fit and contextual appropriateness. Their written diagnostic performance showed uneven ability to apply these criteria to a concrete AI-generated curriculum plan. They often recognised visible implementation problems, while less consistently diagnosing assessment quality, factual verification, author responsibility and objective-task-evidence alignment.

This pattern suggests that AI-assisted learning may produce awareness of responsible AI use before it produces stable professional judgement. Students may learn to say that AI outputs require human review, while still struggling to perform that review at the level expected in vocational practice. The problem extends beyond AI literacy and concerns the gap between declarative judgement language and situated diagnostic action.

This point is consistent with research in educational assessment and L2 writing showing that observable performance can reflect interactions among cognitive, affective, motivational and self-regulatory processes [46]. In the present case, students’ final AI-assisted documents alone could not show whether such processes had developed into stable professional judgement. The no-AI diagnostic task therefore elicited what students could evaluate independently when AI support was unavailable.

AI-accelerated drafting may intensify this gap. Students may experience fast improvement in document quality without internalising the reasoning that makes improvement possible. Teacher feedback can correct specific draft problems, but transfer of judgement to a new case still requires deliberate practice. The no-AI diagnostic task is therefore important because it separates students’ ability to talk about AI judgement from their ability to enact judgement when AI support is unavailable.

### 5.4 Implications for vocational education and professional capability development

The findings suggest that AI-output judgement should become an explicit learning outcome in vocational programmes. In fields where students produce plans, proposals, service scripts, designs, reports or client-facing documents, generative AI can supply the surface form of professional work. Vocational teaching therefore needs to shift part of its attention from output production to output diagnosis.

One practical strategy is to require judgement logs alongside AI-assisted assignments. Such logs can ask students to record what AI generated, which parts they accepted, which parts they questioned, what professional criterion guided revision and what human responsibility remained. A second strategy is diagnostic assessment: students can be asked to identify problems, justify reasoning and propose improvements in flawed AI-generated outputs, especially without AI access. A third strategy is embodied or practice-based testing. Simulation, role play, field enactment or workplace-based review can expose whether a written plan works when real users, learners, clients, tools, time limits and safety constraints are involved.

The study also suggests that vocational teachers need support for their own calibration work. Teacher calibration requires field expertise, rapid diagnosis and the ability to translate professional criteria into learnable feedback. Professional development should therefore extend beyond training teachers to use AI tools and include how to design diagnostic tasks, judge AI-generated professional outputs and teach students to take responsibility for AI-assisted work.

These implications point to a broader redesign principle for vocational education. Generative AI can be treated as part of the ordinary production environment for student work, while students must learn to judge what AI produces. The central question for vocational programmes is how to cultivate graduates who use AI productively while retaining responsibility for professional quality, contextual fit and human consequence.

## 6. Conclusions

This study examined how generative AI reshaped task-based vocational learning in a curriculum design course and how students’ judgement of AI-generated professional work could be made visible. The case shows that when AI can produce complete-looking assignment drafts within seconds, the educational value of a vocational task depends increasingly on the judgement surrounding that document. Students need to identify what is missing, explain why it matters and revise the output through domain-specific professional criteria.

The findings have three implications. First, the stated-applied judgement gap is a useful pattern for describing how students may articulate responsible AI-use principles while applying those principles unevenly to an AI-generated professional output. Second, no-AI diagnostic assessment can help examine students’ AI-output judgement when completed task documents can be produced through generative AI. Third, teacher calibration can support classroom revision by making professional judgement criteria visible during students’ work with AI-assisted drafts.

The study has clear limitations. It is an interpretive single-case study involving one course, one teacher and one cohort of 39 vocational students. The diagnostic task was a structured elicitation device and should not be treated as a standardised measure of AI competence. The study also lacked a pre-course baseline, control group and systematic record of students’ AI interaction histories. Coding was conducted by the instructor-researcher, and no formal independent inter-coder reliability statistic was calculated. The supplied codebook, segmentation rules and anonymised dataset should therefore be read as transparency supports for an interpretive analysis, not as evidence of measurement reliability. The study’s contribution lies in offering a contextually grounded account of how AI-output judgement can be taught, elicited and analysed in a real vocational classroom.

Future research could examine similar diagnostic tasks across vocational domains with different degrees of risk, procedural certainty and design ambiguity. Longitudinal studies could also investigate whether judgement logs, annotated AI drafts, teacher calibration routines and practice-based testing improve students’ transfer of AI-output judgement across tasks and workplace contexts. Overall, the study reframes AI-assisted vocational learning as a problem of professional judgement: vocational capability now includes productive AI use and the capacity to judge what AI produces.

## Supporting information

S1 Appendix Study tour curriculum design: AI-generated plan diagnosis and learning reflection record. This file provides the English translation of the course-based reflective assessment and no-AI diagnostic instrument used in the study.
(DOCX)

S2 Appendix Coding framework and judgement-level criteria. This file provides the segmentation rules, thematic coding structure, claim-level codebook, holistic written judgement-level rubric and anonymisation procedures used in the analysis.
(DOCX)

S1 Data Minimal anonymised dataset underlying the descriptive findings. This file provides the de-identified student-level judgement levels, claim-level coding data, aggregate coding tables and anonymised excerpts used to support the results reported in Tables 1 and 2.
(XLSX)
