# Supplementary material for: Evaluating AI-generated curriculum designs in vocational education: Evidence from a No-AI diagnostic assessment
Source: PLoS One. 2026 Aug 14;21(8):e0352555. doi: 10.1371/journal.pone.0352555 (PMC13475960; doi:10.1371/journal.pone.0352555)
Supplement: Supplementary file 1 [file pone.0352555.s001.docx]

S1 Appendix. Study Tour Curriculum Design: AI-Generated Plan Diagnosis and Learning Reflection Record

*English Translation of the Course-Based Reflective Assessment Instrument*

**Note.** This document is an English translation of the reflective assessment instrument used in the course. The original student responses were written in Chinese.

# Part One: How You Used AI in This Course

**1.** When did you start using AI tools relatively frequently? Which AI tools do you mainly use?

**2.** In the assignments for Study Tour Curriculum Design, what content did your group mainly ask AI to help with?

**3.** Among all the assignments completed by your group, which parts best reflected your own ideas or judgement? You must provide three concrete examples.

**4.** After the teacher discussed your plan with your group, what changes were made to your plan? Please write down the three changes that impressed you most.

# Part Two: “Being Able to Use AI” and “Being Able to Judge AI”

**1.** When using AI to generate assignments for this course, did you actively check whether the content provided by AI was reasonable? What did you usually check?

**2.** After learning in this course, do you feel that you are better able to identify problems in AI-generated study tour plans? Why?

**3.** If you work in the future as a study tour instructor, curriculum designer, or tourism education practitioner, what work do you think AI can complete for you? What work must be judged by humans?

**4.** In one sentence, write the difference between “being able to use AI” and “being able to judge AI-generated content”.

**5.** Looking back from when you started using AI in your first year to the end of your second year, how has your ability to judge AI-generated content improved? Please explain in detail.

# Part Three: Diagnose the Following AI-Generated Study Tour Curriculum Plan

The following is an excerpt from an AI-generated study tour curriculum plan. It appears relatively complete, but it may contain problems in curriculum design. You must identify five problems. For each problem, clearly write: where the problem is; why it is a problem; and how you would revise it. You may analyse it from aspects such as target school stage, curriculum objectives, study tour tasks, on-site implementation, learning outcomes, assessment methods, safety management, and historical fact verification.

## AI-Generated Plan Excerpt

**Course theme:** Entering Luoyi Ancient City and Inheriting a Thousand Years of Culture **/ Target learners:** Third-grade primary school students

**Study tour duration:** 45 minutes **/ Study tour sites:** Luoyi Ancient City, Wenfeng Pagoda, Prefectural Confucian Temple, Ancient City Wall, and Grand Canal-related heritage sites

## Curriculum Objectives

Understand Luoyang’s profound historical culture and Grand Canal commercial civilisation.

Master the formation mechanisms of urban spatial structure and commercial networks during the Sui and Tang dynasties.

Enhance students’ cultural confidence and improve their comprehensive practice ability, creativity, and teamwork ability.

Cultivate students’ love for excellent traditional Chinese culture through study tour activities.

## Activity Procedure

**Step 1.** The teacher leads students to visit Luoyi Ancient City, listen to a guide’s introduction to Luoyang’s history and culture, and take photos of content they find interesting.

**Step 2.** Students freely form groups to interview tourists, learn about tourists’ views of the traditional culture of Luoyi Ancient City, and search for related information on mobile phones.

**Step 3.** Students hold group discussions on the Grand Canal, Tang sancai ceramics, tea culture, Hanfu culture, ancient architecture, and intangible cultural heritage techniques, and analyse the development characteristics of ancient commercial civilisation in Luoyang.

**Step 4.** Each group produces a study tour poster to present its understanding of Luoyang’s traditional culture.

**Step 5.** Each group gives a three-minute presentation, and the teacher conducts a comprehensive evaluation based on students’ performance.

## Assessment Method

The teacher evaluates students in terms of participation, teamwork, cultural understanding, creative expression, and comprehensive literacy.

## Safety Requirements

Students should pay attention to safety during the study tour, follow the teacher’s arrangements, and visit in a civilised manner.

# Your Diagnosis

**Problem One:**

Reason:

Revision suggestion:

**Problem Two:**

Reason:

Revision suggestion:

**Problem Three:**

Reason:

Revision suggestion:

**Problem Four:**

Reason:

Revision suggestion:

**Problem Five:**

Reason:

Revision suggestion:
