# Supplementary material for: Evaluating AI-generated curriculum designs in vocational education: Evidence from a No-AI diagnostic assessment
Source: PLoS One. 2026 Aug 14;21(8):e0352555. doi: 10.1371/journal.pone.0352555 (PMC13475960; doi:10.1371/journal.pone.0352555)
Supplement: Supplementary file 2 [file pone.0352555.s002.docx]

**S2 Appendix. Coding Framework for Student AI-Output Judgement**

**Coding Framework for Student AI-Output Judgement**

This appendix provides the coding framework used to analyse students' handwritten responses in the no-AI diagnostic task. It is designed to clarify the segmentation rules, coding categories, judgement-level rubric, and counting procedures used for Tables 1 and 2 in the manuscript.

# 1. Scope of the Coding Framework

The framework was applied to the closed-book handwritten assessment administered at the end of the Study Tour Curriculum Design course. Parts One and Two were used to identify students' stated AI-use practices, AI judgement criteria, and projected human-AI division of labour. Part Three was used to analyse how students diagnosed a concrete AI-generated study tour curriculum plan without access to AI tools.

The appendix distinguishes between two analytic layers. The first layer codes distinct diagnostic claims in Part Three using twelve problem categories (CJ1-CJ12). The second layer assigns each student's full Part Three response to an overall written judgement level from 0 to 4. Claim-level coding records what types of problems students noticed; holistic level assignment records the depth and integration of each student's diagnostic reasoning.

# 2. Segmentation and Counting Rules

Unit of analysis. A distinct diagnostic claim was defined as a meaningful diagnostic unit, usually consisting of a problem identified by the student, the reason given for that problem, and the proposed modification. A claim could be a full written entry or part of a longer entry if the student addressed more than one analytically distinct issue.

Claim retention. The prompt invited each of the 39 students to identify five problems, creating 195 potential entries. During coding, entries that repeated the same point within a student's response were merged, and entries that named a problem without a substantive diagnostic claim were excluded from claim-level counting. This process retained 182 distinct diagnostic claims for coding.

Multiple coding. A single diagnostic claim could receive more than one code when it addressed multiple dimensions. For example, a statement about primary school third-graders completing interviews at multiple sites within forty-five minutes could be coded as learner population appropriateness, operational feasibility, and safety management adequacy.

Student-level presence. In category-level reporting, student-level presence was counted once per student per category, regardless of how many claims the student made in that category. Diagnostic-claim counts record the number of coded units assigned to each category. Because multiple coding was allowed, diagnostic-claim counts are not additive across categories.

# 3. Thematic Coding for Parts One and Two

Parts One and Two were analysed inductively to identify recurring themes in students' accounts of AI use, AI judgement, and future professional human-AI division of labour. The table below summarises the thematic coding structure used for these sections.

| **Theme** | **Initial codes included** | **Analytic focus** | **Use in manuscript** |
| --- | --- | --- | --- |
| AI-normalised assignment production | AI as draft generator; AI as source of structure; AI as formatting tool; AI as language refinement tool | How students reported using AI in course assignments and how AI entered the assignment production process | Supports RQ1 context and RQ3 interpretation |
| Stated AI judgement criteria | Checking factual accuracy; checking feasibility; checking learner appropriateness; checking alignment with teacher requirements; checking safety | How students described the difference between using AI and judging AI-generated content | Supports RQ3 and interpretation of stated criteria |
| Projected professional responsibility | Human review of routes; site-specific judgement; safety decisions; factual verification; teacher/professional responsibility | How students imagined human-AI task division in future study tour, curriculum design, or tourism education work | Supports RQ3 discussion of future professional vision |

# 4. Claim-Level Codebook for Part Three

The twelve coding categories were developed from the course's explicit curriculum design criteria, the diagnostic prompt, and the theoretical focus on evaluative and professional judgement. The framework was refined after preliminary reading of the first ten responses to clarify category boundaries.

| **Code** | **Problem category** | **Operational definition** | **Typical indicators and decision boundaries** |
| --- | --- | --- | --- |
| CJ1 | Learner population appropriateness | Judgements about whether the target learners' age, prior knowledge, developmental stage, and learning capacity match the proposed objectives and tasks. | Includes comments that third-grade learners may be unable to understand abstract historical concepts, conduct independent interviews, use phones, or complete complex group tasks. Excludes comments focused mainly on time, route, or site sequence unless learner fit is explicitly discussed. |
| CJ2 | Site and resource selection judgement | Judgements about the number, location, sequence, educational relevance, and resource fit of study tour sites or materials. | Includes comments on excessive sites, weak connection among selected sites, unclear resource logic, or unsuitable resource combinations. Excludes pure timing concerns unless the site choice itself is criticised. |
| CJ3 | Learning objective quality | Judgements about whether objectives are specific, feasible, age-appropriate, and aligned with the course theme and tasks. | Includes comments that objectives are too broad, too abstract, unrealistic, poorly sequenced, or mismatched with tasks. Excludes comments on task design alone unless objective-task alignment is addressed. |
| CJ4 | Task educational validity | Judgements about whether activities generate learner action, observation, inquiry, expression, reflection, or meaningful engagement. | Includes comments that students only listen passively, take photos without inquiry, discuss too many unrelated topics, or complete tasks lacking educational purpose. Excludes general feasibility concerns unless the educational value of the task is discussed. |
| CJ5 | Operational feasibility | Judgements about practical implementability, including time allocation, route sequence, workload, required materials, and on-site management. | Includes comments that forty-five minutes is too short, the itinerary is overloaded, group reporting is unrealistic, or the activity sequence cannot be implemented. Excludes safety-specific concerns unless implementability is the main issue. |
| CJ6 | Safety management adequacy | Judgements about whether safety arrangements are specific, age-appropriate, sequenced properly, and adequate for real field implementation. | Includes comments on vague safety rules, lack of pre-activity safety briefing, street risks, narrow paths, crowd control, interviews with strangers, or phone use by young pupils. Excludes general discipline comments unless safety risk is explicit. |
| CJ7 | Learning evidence quality | Judgements about whether outputs or performances can show that learning has occurred. | Includes comments on whether photos, posters, reports, questions, or presentations provide observable evidence of learning. Excludes general task comments that do not address evidence or output quality. |
| CJ8 | Assessment operationalisability | Judgements about whether evaluation criteria are concrete, observable, age-appropriate, and aligned with learning tasks and outputs. | Includes comments that evaluation is vague, relies on broad qualities such as participation or comprehensive literacy, or lacks observable criteria. Excludes comments on learning outcomes unless assessment criteria are addressed. |
| CJ9 | Fact verification | Attention to the accuracy, source-checking, and verification of historical, cultural, geographical, or site-specific information. | Includes comments on verifying historical claims, checking the relation between sites and topics, and using reliable sources. Excludes general content richness comments without source or accuracy concerns. |
| CJ10 | AI judgement metacognition | Explicit awareness that AI-generated content requires checking, questioning, revision, or human review. | Includes statements that AI plans look complete but require scrutiny, that AI may hallucinate, or that AI outputs need comparison with reality. Excludes ordinary task critique that does not mention AI judgement or checking. |
| CJ11 | Teacher scaffolding role recognition | Recognition of teacher guidance, facilitation, questioning, or professional correction as necessary for improving AI-generated or student-designed plans. | Includes comments that teachers should set questions, guide observation, provide prompts, structure discussion, or correct student tasks. Excludes teacher evaluation comments unless facilitation or scaffolding is central. |
| CJ12 | Curriculum author responsibility | Statements showing that the human designer remains responsible for whether an AI-assisted curriculum is safe, feasible, accurate, and educationally meaningful. | Includes comments assigning responsibility to the curriculum designer or study tour professional for safety, accuracy, learner fit, and implementation. Excludes narrow comments on one technical defect unless broader responsibility is expressed. |

# 5. Holistic Written Judgement Level Rubric

After claim-level coding, each student's complete Part Three response was assigned an overall written judgement level. This was a holistic interpretive judgement based on the range of problems identified, the reasoning provided, and the degree of integration across curriculum design criteria. The levels were used to describe patterns in written diagnostic performance and were not treated as standardised psychometric scores.

| **Level** | **Descriptor** | **Indicative features** |
| --- | --- | --- |
| Level 0 | No substantive diagnostic engagement | Blank, irrelevant, or non-diagnostic response; no meaningful problem identification or reasoning. |
| Level 1 | Minimal diagnostic engagement | Isolated or vague comments; limited explanation; modification suggestions are absent, generic, or unrelated to curriculum design logic. |
| Level 2 | Surface checker | Recognition of visible implementation problems such as time pressure, learner age mismatch, site overload, task difficulty, or safety insufficiency; reasoning remains mainly local and practical. |
| Level 3 | Partial curriculum judge | Identification of multiple substantive problems with some educational design reasoning; connections among objectives, tasks, evidence, assessment, and learner population are present but uneven. |
| Level 4 | Curriculum logic judge | Integrated judgement across learner population, objectives, tasks, learning evidence, assessment, feasibility, safety, and responsibility; critiques link visible problems to deeper curriculum logic and propose actionable modifications. |

# 6. Translation, Anonymisation, and Audit Trail

Student responses were handwritten in Chinese and transcribed into typed Chinese text for coding. The typed transcriptions were checked manually against the original handwritten sheets. Excerpts reported in English were translated by the instructor-researcher and checked against the original responses for semantic accuracy.

Student identifiers were replaced with codes S01-S39 before analysis and reporting. The appendix contains no personally identifiable information and reports only analytic categories, decision rules, and de-identified examples of coding logic.
